# Targeting the ileal bile salt transporter in the treatment of non-alcoholic fatty liver disease

**DOI:** 10.1007/s12072-021-10134-5

**Published:** 2021-02-01

**Authors:** Peter L. M. Jansen

**Affiliations:** Amsterdam University Medical Center, Meibergdreef 9, 1105 AZ Amsterdam, The Netherlands

Non-alcoholic fatty liver disease (NAFLD) is the liver component of the metabolic syndrome. The disease mainly but not exclusively develops in people who are overweight or obese and in patients with type 2 diabetes. In Western society, NAFLD is highly prevalent and is gaining importance among people in Latin America and Asia [1]. NAFLD can give rise to non-alcoholic steatohepatitis (NASH), cirrhosis and hepatocellular carcinoma. The hypercaloric Western style diet, rich in fructose and trans-fats, is a main cause of the disease, particularly in patients with genetic susceptibility such as carriers of the PNPLA3 polymorphism [2]. The majority of patients with NASH have insulin resistance suggesting that this plays a role in the pathophysiology of the disease. Although changes in lifestyle such as increased mobility and dietary adaptations are key therapeutic interventions in patients with NASH, caretakers of these patients know that adherence to these lifestyle changes is difficult in the long run. Therefore, effective drugs are needed to treat patients with NASH and prevent progression of the disease.

In search for therapeutic targets, investigators realized that bile salts may play a role in the development of NASH. Serum and intrahepatic bile salt levels in patients with NASH are increased and regulatory mechanisms to prevent bile salt-induced hepatotoxicity are disturbed [3, 4]. Bile salts in high concentrations are hepatotoxic and pro-inflammatory. Therefore, bile salts and bile salt receptors seem to be a logical target in the treatment of NASH.

**Fig. 1:**
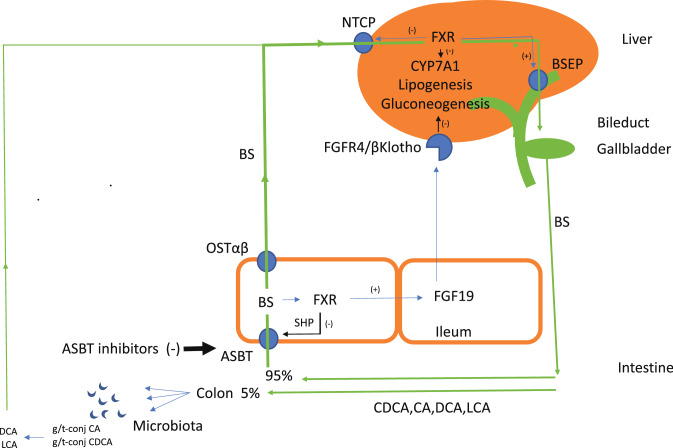
Enterohepatic circulation and regulation of lipogenesis. The reabsorption of bile salts in the ileum is mediated by ASBT (apical sodium-dependent bile salt transporter). Bile salts exit the ileum mucosa cells via OST (organic solute carrier) α/β to enter the portal blood. In the liver bile salt uptake is supported by NTCP (sodium taurocholate co-transporting polypeptide). Bile salt exit from hepatocytes is mediated by BSEP (bile salt export pump). Bile is stored in the gallbladder and bile salts enter the small intestine upon gallbladder contraction. In addition to a function in the digestion of lipids, bile salts also have a signaling function. In intestinal mucosa cells bile salts activate FXR and this induces the synthesis and expression of FGF19 (fibroblast growth factor 19) and SHP (small heterodimer partner). SHP is a transcriptional regulator that reduces the expression of ASBT. This serves as feed-back regulation, reducing bile salt absorption. FGF19 expression is stimulated by binding of bile salts to FXR. FGF19 is secreted into the portal blood. In the liver FGF19 binds to the FGFR4/βKlotho complex. This activates a number of signaling proteins that cause the downregulation of CYP7A1 and NTCP and upregulation of BSEP. In addition, FGF19 suppresses hepatic lipogenesis and gluconeogenesis. Inhibition of ASBT increases the excretion of bile salts into the colon. This reduces the serum bile salt concentration and reduces the circulating bile salt pool. It also decreases the synthesis of FGF19 in the ileum. The increased excretion of bile salts to the colon stimulates chloride and water secretion by the colon, changes the intestinal microflora and increases the production of the secondary bile salts deoxycholate and lithocholate. Lithocholate is a potent ligand for TGR5 (Takeda G-protein coupled bile salt receptor). This stimulates TGR5-mediated energy metabolism, the therapeutic value of which needs to be established

The traditional role of bile salts is in the digestion of dietary fats. Bile salts enable pancreatic lipase in the small intestine to hydrolyze triglycerides to mono- and diglycerides and free fatty acids, molecules that can be absorbed. For this function, bile salts need to be present in the intestine in millimolar concentration. Therefore, bile salt metabolism needs to be tightly regulated, not only to avoid bile salt overload but also bile salt depletion. Ninety-five percent of bile salts secreted into the small intestine is reabsorbed (Fig. 1). This protects the organism from bile salt depletion. Conjugated bile salts are reabsorbed in the terminal ileum, uncharged unconjugated bile salts can be reabsorbed by non-carrier mechanisms in jejunum and ileum [5]. Reabsorption of conjugated bile salts in the ileum is mediated by the apical sodium-dependent bile salt transporter ASBT (ileum sodium-dependent bile salt transporter) [6]. ASBT is located in the apical membranes of ileum mucosa cells. From the mucosa cells bile salts are delivered to the portal blood by the action of the organic solute carrier OSTα/β in the basolateral cell membrane domain [7]. During transcellular passage, bile salts interact with the nuclear hormone receptor FXR (farnesoid X-receptor) [8]. FXR is a transcriptional regulator of FGF19 (fibroblast growth factor 19) and SHP (small heterodimer partner). In the intestine SHP acts as a transcriptional regulator to suppress ASBT expression. In the liver, SHP functions in the FXR-mediated down-regulation of CYP 7A1 and the bile salt uptake carrier NTCP (sodium taurocholate co-transporting polypeptide).

Upon bile salt absorption, FGF19 synthesis in the intestinal mucosa cells is activated. FGF19 is released into the portal blood and delivered to the liver to bind to the FGFR4/βKlotho receptor complex [8]. Through the action of the signaling proteins JNK and ERK, this leads to down-regulation of CYP7A1 transcription, the enzyme that plays a key role in the conversion of cholesterol to the primary bile salts chenodeoxycholic acid (CDCA), cholic acid (CA) and muricholic acid (MC, in mice). A non-CYP7A1-dependent alternative pathway, supporting the synthesis of CDCA and MC, is not affected. Bile salts are conjugated in the liver with either glycine or taurine, transported across the canalicular membrane by the bile salt export pump (BSEP) and delivered to the gallbladder. From here, bile salts are secreted into the intestine. Part of the bile salt pool recirculates and is reabsorbed in the ileum, taken up in the liver by NTCP and secreted into bile by BSEP, thus closing the circle.

CYP7A1 is a tightly regulated hepatic enzyme. Its main action is the conversion of cholesterol to bile salts, thereby compensating for the 5% loss of bile salts that escape from reabsorption. Through the action of hepatic FXR and SHP, CYP7A1 transcription and CYP7A1 protein expression are down-regulated, thus preventing bile salts to reach hepatotoxic levels. In healthy persons, the FXR-mediated down-regulation of CYP7A1 serves in the feed-back regulation of bile salt synthesis. In NASH, these regulatory mechanisms are disturbed causing inappropriately high intrahepatic and serum bile salt levels [4].

Recent studies showed that bile salts through the action of FXR, FGF19 (fibroblast growth factor 19) and TGR5 (Takeda G-protein coupled bile salt receptor) regulate energy metabolism, bile salt synthesis, lipid oxidation, lipogenesis and gluconeogenesis [9]. These metabolic interrelations drew the attention of drug developers and led to the synthesis of novel therapeutic agents, with the FXR-ligand obeticholic acid (7ethyl-CDCA) as a leading compound. However, the full advantage of these new insights still needs to be established. For instance, in two phase III studies, it became apparent that obeticholic acid although successful in reducing fibrosis, inflammation and steatosis in NASH patients, increased LDL- and reduced HDL-cholesterol, a disadvantage in a predominantly obese population [10, 11]. Increased mortality in OCA-receiving patients with primary biliary cholangitis with advanced fibrosis led to the recommendation not to use this drug in patients with F4 fibrosis and cirrhosis (FDA black box warning).

The association between bile salts and metabolism is complex and the interplay of bile salts, lipids and glucose can be approached from different angles, some pathways appear more promising than others. We now have steroidal and non-steroidal FXR agonists, agents that sequester bile salts in the intestine and drugs that inhibit ASBT [12, 13]. Other potential drugs for the treatment of NASH include FGF19-mimetics, FGF21 derivatives and agents that target the bile salt receptor TGR5. TGR5 is expressed in many tissues and organs including brown adipose tissue, cholangiocytes, brain and colon [14–17]. In addition, agents have been developed that interact with PPAR γ (peroxisome proliferator-activated receptor gamma, the ‘glitazone receptor’ in adipose tissue) and PPAR α (regulator of lipid metabolism in the liver) [18].

In this issue of Hepatology International, Japanese investigators report on the effect of the ASBT inhibitor elobixibat in mice with methionine choline-deficient (MCD) diet-induced NAFLD/NASH [12]. These mice have a fatty liver, increased inflammatory parameters, mild pericentral fibrosis and elevated serum bile acid levels. In the experimental model used by the authors, BL/6N mice received a MCD diet for 8 weeks. In half of these mice, the MCD diet was combined with elobixibat during the last 4 weeks looking for a preventive effect of elobixibat. The main findings of this relatively short study are a reduction of inflammatory parameters in the treated group, less fibrosis and reduced serum bile salts and alterations of the gut microflora as compared to the non-treated group. There was no effect on hepatic steatosis.

The mechanism of action of ASBT inhibitors in NASH is not completely understood. ASBT inhibitors have an interesting developmental history. Initially, they were advanced as candidates for the treatment of hypercholesterolemia. Irritable bowel syndrome and chronic constipation are other indications. This action is based on the fact that ASBT inhibitors increase the bile salt concentration in the colon and so stimulate chloride and water secretion and alter the intestinal microbiota. By doing this, they soften the stools and stimulate colonic motility. For NASH, ASBT inhibitors are under investigation both in animal studies and in human phase II trials. A recently published study reported the effect of the ASBT inhibitor volixibat in patients with NASH [13]. The study was negative in that there was no significant effect on either fibrosis, inflammation or steatosis.

Both volixibat and elobixibat inhibit bile acid absorption in the terminal ileum. By doing this, these drugs lower the circulating bile salt pool but by inhibiting bile salt reabsorption they also reduce the synthesis and release of FGF19. Lowered FGF19 secretion impairs the physiological feed-back inhibition of CYP7A1 in the liver and reduce the beneficial effects of natural FGF19 on lipidogenesis and gluconeogenesis. In the volixibat study, an increase in 7alpha-hydroxy-4-cholesten-3-one (C4, a serum biomarker of bile salt synthesis) was observed as indication of increased bile salt synthesis in the liver due to a lowered activity of FGF19.

Other targets, such as an increased bile salt concentration in colon and stool and changes of the microbial colonic flora, were achieved in the Japanese study [12]. However, steatosis was not affected. Lipotoxicity plays an important role in the pathogenesis NASH and therefore the lack of effect on steatosis may be a disadvantage of these drugs. Decreased FGF19 expression may underly the lack of effect on steatosis by these drugs and this may be a stumbling block for the development and clinical application of these agents.
